# Phase Transition Effects on Mechanical Properties of NIPA Hydrogel

**DOI:** 10.3390/polym10040358

**Published:** 2018-03-23

**Authors:** Ni Zhang, Shoujing Zheng, Zhouzhou Pan, Zishun Liu

**Affiliations:** International Center for Applied Mechanics, State Key Laboratory for Strength and Vibration of Mechanical Structure, Shaanxi Engineering Research Center of Nondestructive Testing and Structural Integrity Evaluation, Xi’an Jiaotong University, Xi’an 710049, China; nizhang961228@xjtu.edu.cn (N.Z.); zsjaa123@stu.xjtu.edu.cn (S.Z.); panzhouzhou@stu.xjtu.edu.cn (Z.P.)

**Keywords:** temperature-sensitive hydrogel, phase transition, mechanical properties, cyclic load, toughening

## Abstract

Due to its excellent temperature sensitivity, the Poly(*N*-isopropylacrylamide) (NIPA) hydrogel has attracted great interest for a wide variety of applications in tissue engineering and regenerative medicine. NIPA hydrogel undergoes an abrupt volume phase transition at a lower critical solution temperature (LCST) of 30–35 °C. However, the mechanical behaviors of NIPA hydrogel induced by phase transition are still not well understood. In this study, phase transition effects on mechanical properties of NIPA hydrogel are quantitatively studied from experimental studies. The mechanical properties of NIPA hydrogel with the LSCT around 35 °C are systemically studied with varying temperatures (31–39 °C) under a tensile test. We find that the mechanical properties of NIPA hydrogel are greatly influenced by phase transition during the tension process. The maximum nominal stress and maximum stretch above the LCST are larger than those of below the LCST. The Young’s modulus of NIPA hydrogel is around 13 kPa at 31 °C and approximately 28 kPa at 39 °C. A dramatic increase of Young’s modulus values is observed as the temperature increases through the phase transition. The samples at a temperature around the LCST are easy to rupture, because of phase coexistent. Additionally, NIPA hydrogel displays toughening behavior under a cyclic load. Furthermore, the toughening characteristic is different between the swollen state and the collapsed state. This might originate from the internal fracture process and redistribution of polymer chains during the tension process.

## 1. Introduction

Intelligent hydrogels have attracted considerable attention because of their abilities to change their volumes and properties in response to external stimuli (temperature, pH, light, ionic strength, electric fields, magnetic fields) [1,2,3,4]. Among all stimuli-sensitive hydrogels, poly(*N*-isopropylacrylamide) (NIPA) is one of the widely studied hydrogels due to its excellent temperature sensitivity. It undergoes an abrupt volume phase transition at a lower critical solution temperature (LCST). From experimental study, it can be observed that the LCST lies between 30 and 35 °C, and the exact temperature is a function of the detailed microstructure of the macromolecule [5]. The hydrophilic moieties (–CONH–) may interact with water molecules through hydrogen bonding below the LCST, which leads to water absorption by the hydrogel. Therefore, the hydrogel is swollen and hydrophilic. However, as the external temperature increases, the hydrogen—bonding interactions become weakened or destroyed. Thus, the hydrophobic interactions among the hydrophobic moieties (–CH(CH_3_)_2_) grow to be strong, which induces the freeing of the entrapped water molecules from the network. When the temperature is above the LCST, the hydrogel shrinks (or undergoes deswelling) and turns into a collapsed and hydrophobic state expelling water, due to the hydrophobic interactions becoming dominant. Combined with water release, polymer chains collapse abruptly and the phase separation of the NIPA hydrogel system occurs. Phase transition induced by uniaxial tensile force is also observed by experimental phenomenon near the LCST [6]. Following this, Suzuki & Ishii [7] also observed phase coexistent in NIPA hydrogel around the LCST under mechanical constraints. Due to an abrupt volume phase transition that is sensitive to temperature and tensile force sensitivity, NIPA hydrogels have attracted great interest for a wide variety of applications, such as tissue engineering [8,9] and drug delivery [10,11]. However, the effect of phase transition on the mechanical properties of NIPA hydrogel is still not well understood. One reason for the lack of information might be ascribed to the difficulties in clamping the hydrogel in order to retrieve the mechanical properties, due to it being extremely fragile [12].

Although some mechanical properties of NIPA hydrogel are experimentally studied, the works were mostly concentrated on a special NIPA gel condition. For example: in a work on NIPA gel functionalized with ionic groups, Gundogan et al. [13] firstly obtained that the Young’s modulus under compression is about 5.63 kPa. The only partial information about the tensile modulus of pure NIPA was indirectly derived from studies on the latex/NIPA composite and is affected by the significant polyester influence on the mixture mechanical properties [14]. Matzelle et al. [15] proposed scan force microscopy (SFM) as a new technique for the study of NIPA hydrogel, obtaining a shear modulus between 5 and 10 kPa. Tensile experiments performed for the first time on the pure material evidenced a rupture limit for a strain around 30% with a Hookean modulus of 24.8 kPa and neo-hookean modulus of 7.3 kPa by Puleo et al. [16]. However, the above-mentioned mechanical properties of NIPA hydrogel are mainly studied at room temperature. Systemic studies at temperatures through the LCST are still lacking. 

Regarding the phase transition effect on mechanical properties, a few researches have examined the phase transition under mechanical constraints in theoretical studies [17,18]. Cai & Suo [17] explained the phenomenon thermodynamically. Zheng and Liu [18] proposed a theoretical method that can predict the critical stress which triggers phase transition under a monotonic load. However, no one has investigated the effects of phase transition on mechanical properties in experimental tests. Furthermore, in practical applications, more works focus on the mechanical properties under a cyclic load. Therefore, before we adopt the gel materials into wider application fields, it is very essential for us to understand the mechanical properties under a cyclic load, especially quantitatively providing information for the application in tissue engineering.

In this study, we aim to quantitatively study the mechanical properties of NIPA hydrogel, as well as the phase transition effect on mechanical properties under mechanical constraints and temperature stimuli. In Section 2, a series of quantitative experiments are conducted using a tensile test under a monotonic load and cyclic load at temperatures through the LCST. In Section 3, the mechanical properties and phase transition effect are analyzed in terms of maximum stress, maximum stretch, and the Young’s modulus, which are defined in 10% deformation. In Section 4, evolution characteristics of stress-stretch curves over cycles are shown and evolution of the energy release rate over cycles is discussed, combining the internal fracture process and redistribution of polymer chains during the tension process. Through finding new phenomenon from tests, some conclusions are drawn.

## 2. Experiments

### 2.1. Sample Preparation

Poly(*N*-isopropylacrylamide) gel, a kind of common temperature sensitive hydrogel, is synthesized by a free radical copolymerization [7]. The main synthesis reagents are *N*-isopropylacrylamide (NIPA, monomer), *N*,*N*′-methylenebisacrylamide (BIS, crosslinker), *N*,*N*,*N*′,*N*′-tetramethylethylenediamine (TEMED, accelerator), and ammonium persulfate (APS, initiator). The synthetic scheme is modified after Suzuki & Ishii [7]. In the synthesis, first, 7.8 g of purified NIPA monomer and 133 mg of *N*,*N*′-methylenebisacrylamide (BIS, crosslinker) are dissolved in 100 g deionized water. Next, the prepared solution in the first step is kept in the refrigerator at 4 °C for 8 h to totally dissolve. Thirdly, 240 μL of *N*,*N*,*N*′,*N*′-tetramethylethylenediamine (TEMED, accelerator) is added to the totally dissolved solution. Then, 40 mg of ammonium persulfate (APS, initiator) is added to initiate the reaction. Finally, the mixture is poured into a pre-cleaned glass mould with a 1 mm silicone spacer. It should be noted that the mixture must be immediately poured into a glass mould to avoid the NIPA monomer being separated, if the room temperature is high. Gelation is carried out at 4 °C in the refrigerator for more than 24 h in order to wait for the reaction completion. After gelation, the 1 mm thick sheet of NIPA gel is slightly removed from the glass sheet and is cut into a 4.5 cm × 3 cm rectangle sheet using a laser cutting machine. Subsequently, the gel sheets are washed in deionized water to get rid of residual chemicals and unreacted monomers. The prepared samples are stored in a box filled with deionized water at room temperature for seven days to equilibrate the hydrogels into the fully swollen state for the next test.

The LCST of self-prepared NIPA hydrogel ranges from 34.5 to 35.2 °C. Due to hysteresis of swelling—deswelling, the LCST displays a little difference between the heating process and cooling process. Moreover, the LCST value will exhibit a little fluctuation under mechanical constraints.

### 2.2. Tensile Test

**Figure 1:**
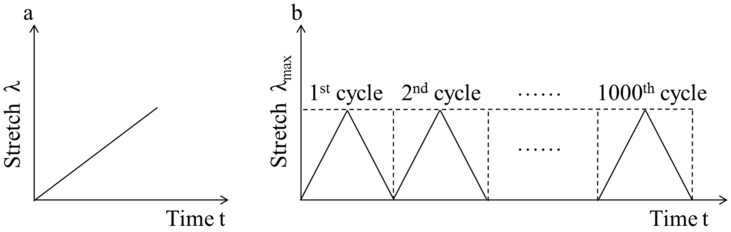
Two types of loading mode ((**a**) monotonic load; (**b**) cyclic load)).

The fully saturated samples are used for all tensile tests. The final sample size is changed into 5.3 cm × 3.6 cm × 0.12 cm in the fully swollen state due to the strong swelling feature of NIPA gel. In the tensile test, the lower side of samples is fixed on the bottom of a water bath and the upper side is clamped using the metallic grippers of a tensile tester (SHIMADZU AGS-X, Shimadzu Corporation, Kyoto, Japan). The sample length between the fixed bottom and the upper gripper is controlled to 1.0 ± 0.05 cm. Before the sample is fixed on the tensile tester, ribbon gauze is glued on the lower and upper sides of the sample to prevent it from breaking or sliding near the gripper during the tension process. We use a load cell of 50 N to apply a uniaxial load to each sample. Under a monotonic load (Figure 1a), the samples are loaded at a rate of 5 mm/min until the samples rupture. Cyclic mechanical tests (Figure 1b) are conducted at a constant cross-head speed (5 mm/min) to a maximum stretch λ_max_ = 1.2. The number of cycles is set to 1000, except for some samples which rupture after less than 1000 cycles. All tests are carried out at different temperatures (31–39 °C) in the water bath. The temperature fluctuation of the water bath is ±0.1 °C. The force and displacement for each sample are recorded by the tensile tester. We plotted the stress-stretch curves of all the samples. The nominal stress is defined as the applied force in the deformed state divided by the cross-sectional area (5.3 cm × 0.12 mm) in the undeformed state. The stretch is the length in the deformed state divided by the length in the undeformed state.

## 3. Mechanical Properties under Monotonic Load

**Figure 2:**
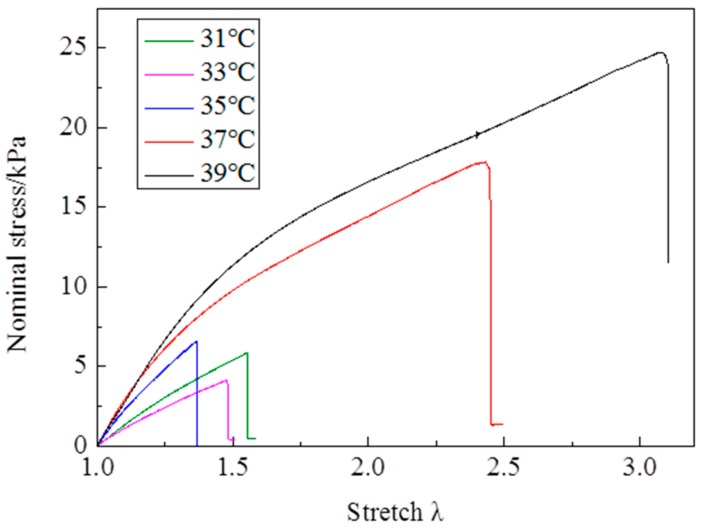
Stress-stretch curves vary with temperatures.

**Figure 3:**
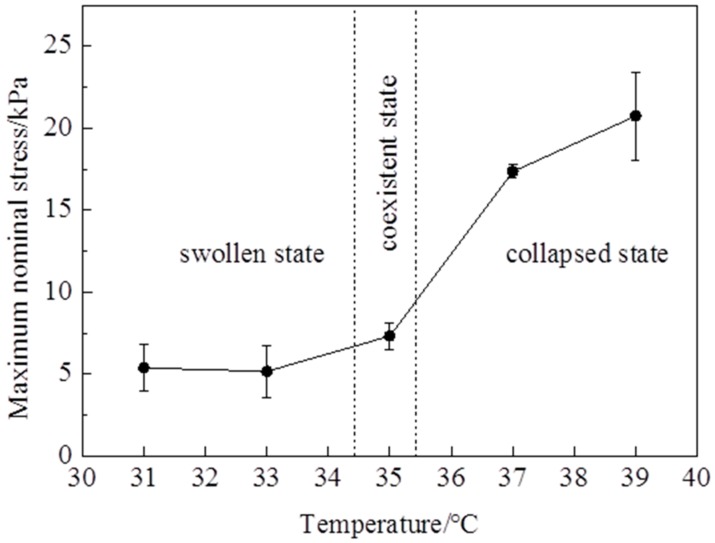
Maximum nominal stress varies with temperatures.

**Figure 4:**
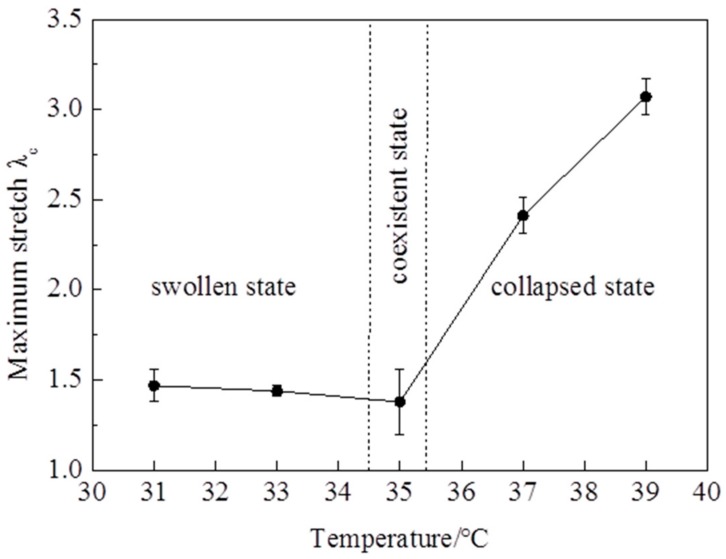
Maximum stretch varies with temperatures.

**Figure 5:**
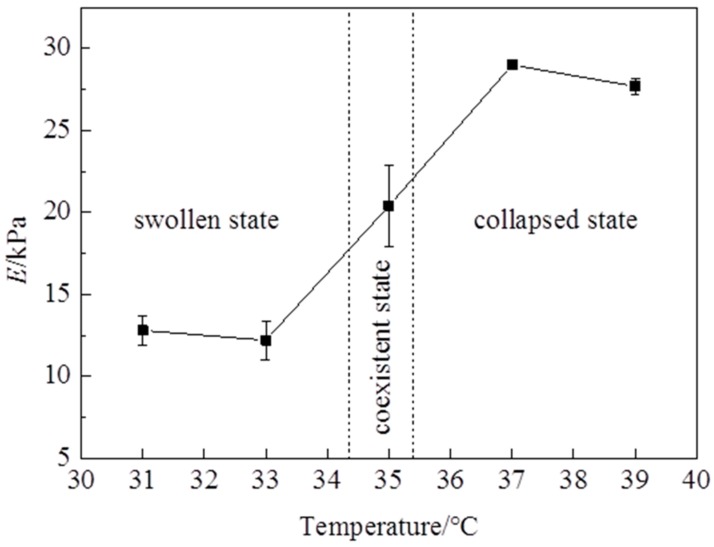
The Young’s modulus *E* varies with temperatures.

The stress—stretch curves of NIPA hydrogel are measured by a tensile test at different temperatures through the LCST at the loading strain rate of 5 mm/min (Figure 2). It is clearly shown that the stress—stretch curves vary with temperatures. With the temperature changing, the hydrogel undergoes a phase transition: the amount of water in the hydrogel in equilibrium varies with the temperature change. Due to the LCST being around 35 °C, the maximum nominal stress and maximum stretch below the LCST are clearly smaller than those of above the LCST (Figure 3 and Figure 4). The maximum nominal stress at 39 °C is around 20 kPa, while the value at 31 °C is only 5 kPa. The critical maximum stretch (λ_c_) at 39 °C can be up to 3, while the λ_c_ at 31 °C is near 1.5. Due to phase discontinuity and non-homogeneous deformation, the samples are much easier to rupture at a rather small stretch at 35 °C. The values of maximum nominal stress and maximum stretch at 35 °C are close to the values below the LCST. The Young’s modulus *E* is calculated from the slope of stress—stretch curves in 10% strain, because NIPA hydrogel is in an elastic state within 10% deformation. A dramatic increase of *E* values is observed as the temperature increases through the phase transition (Figure 5). Furthermore, the Young’s modulus *E* at 39 °C is around 28 kPa, which is twice the value at 31 °C. No special properties are observed in our experiments, except smaller maximum stretch near and below the LCST. 

Under the mechanical constraints, Suzuki et al. [7] argued that stress inhomogeneity along the uniaxial direction is the main reason for the phase coexistence. Furthermore, the ratio of the swollen portion to the total length can be controlled by the degree of elongation during the transition from the collapsed to the swollen phase. According to the conclusions of Suzuki [7], the swollen section induced by tensile force increases with the uniaxial tensile force increasing. Therefore, the critical breaking point should be small near the LCST. Based on thermodynamics, Cai & Suo [17] and Zheng & Liu [18] provided the following stress—stretch relationship:

(1) $$s=NkT\left(\lambda_{3}-V/V_{0}\lambda_{3}{}^{-2}\right)$$

(2) $$f\left(V/V_{0},T\right)=N\nu \left({\left(V/V_{0}\right)}^{-1/3}-{\left(V/V_{0}\right)}^{-1}\right)+\log\left(1-{\left(V/V_{0}\right)}^{-1}\right)+{\left(V/V_{0}\right)}^{-1}+(\chi_{0}-\chi_{1}){\left(V/V_{0}\right)}^{-2}+2\chi_{1}{\left(V/V_{0}\right)}^{-3}=0$$

where *s* is the stress, *N* is the nominal density of the polymer chains, *k* is the Boltzmann constant, *T* is the temperature, λ_3_ is the uniaxial stretch, *V* is the volume of the hydrogel in the current state, and *V*_0_ is the volume of the dry polymer. By combining Equations (1) and (2), it is shown that the stress has two local minima at different volumes in the vicinity of the LCST [17,18]. This furthermore indicates that the mechanical properties near the LCST under mechanical constraint are instable and they exhibit small stretch near the LCST.

The rate—dependent stress—stretch behavior of polyacrylamide—alginate tough hydrogels has recently been studied [19]. The effect of loading rate is also taken into consideration in this study. The mechanical properties at the loading strain rate of 0.01 mm/min are presented in the supplementary information. No significant differences are observed in the stress—stretch curves for NIPA hydrogel at the loading strain rate of 5 mm/min and 0.01 mm/min. However, Zheng & Liu [18] observed the phase transition under mechanical constraints at the loading strain rate of 0.02 mm/min using dynamic mechanical analysis (DMA). Therefore, the loading strain rate may not be one of the factors influencing the expression of phase transition under mechanical constraints. Considering no significant rate dependence for NIPA hydrogel, a constant loading strain rate of 5 mm/min will be used in the following cyclic tests.

## 4. Mechanical Properties under Cyclic Load

The stress—stretch curve of a hydrogel or an elastomer often depends on loading history, such as, double network hydrogels over consecutive loading cycles display softening [20,21,22], because of progressive damage of the polymer networks [21], and tough hydrogel exhibited shakedown over cycles [23]. Here we focus on the evolution of the stress—stretch curves over cycles, and further analyze the phase transition effects on the mechanical properties of NIPA hydrogel.

The tensile experiment, even for little deformations, already produced visible shredding on the material, compromising the possibility to perform cyclic testing. Puleo et al. [16] have pointed out that it was not possible to get any hysteresis measurement. In our study, the mechanical properties of NIPA hydrogel under a cyclic load are performed for the first time. The samples are measured at temperatures of 31–39 °C at the loading strain rate of 5 mm/min. Mechanical cyclic tests are mainly conducted at *λ*_max_ = 1.2. In the preliminary study, hydrogels under a cyclic load would reach a stability state within 2000 cycles [22,23]. Here, the number of cycles is set to 1000. It must be explained that no systemic data at 35 °C is obtained from the tests, because samples in the phase coexistent state are unstable and rather easy to rupture.

**Figure 6:**
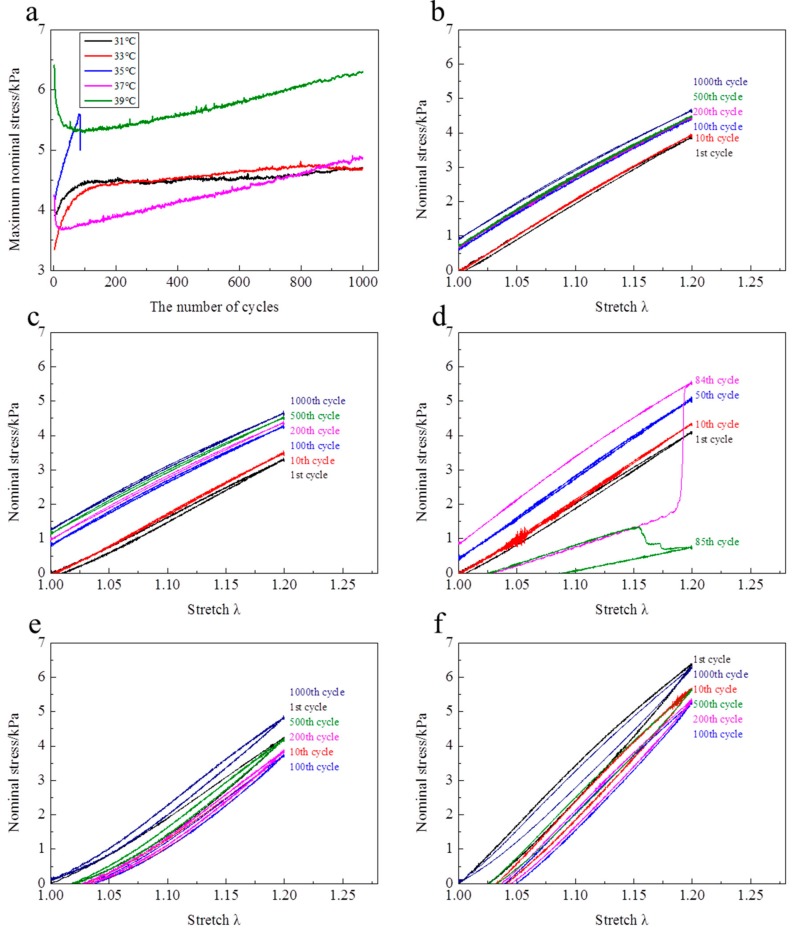
Evolutions of nominal stress over cycles ((**a**) Evolution of maximum stress in each cycle over cycles, and evolutions of stress-stretch curves over cycles at λ_max_ = 1.2 at temperature of (**b**) 31 °C, (**c**) 33 °C, (**d**) 35 °C, (**e**) 37 °C, (**f**) 39 °C).

At λ_max_ = 1.2, the evolutions of stress—stretch curves over cycles at different temperatures are shown in Figure 6. It is clearly observed that the evolution features of stress—stretch curves over cycles above the LCST are totally different from those below the LCST. Below the LCST, the stress—stretch curves increase over cycles (Figure 6b,c). Above the LCST, the stress—stretch curves firstly shakedown over cycles and then increase with cycles increasing (Figure 6e,f). Although the samples at 35 °C rupture within dozens of cycles, the evolution trend is similar with the samples below the LCST. For the evolutions of maximum nominal stress of each cycle over cycles (Figure 6a), they are divided into two stages. Below the LCST (31 and 33 °C), the maximum nominal stresses increase dramatically over cycles to the largest value, and then hardly change over cycles. Above the LCST (37 and 39 °C), the maximum nominal stress firstly reduces rapidly with the cycle increasing to the smallest value, and then linearly increases with cycle increasing. It is worth mentioning that the maximum nominal stress in the 1000th cycle is larger than that in the first cycle at 37 °C. In the first cycle, the hysteresis loops at 37 and 39 °C are clearly larger than the ones at 31 and 33 °C. The hysteresis loops become narrower over cycles at 37 and 39 °C, while the hysteresis loops nearly disappear at 31 and 33 °C. Additionally, a relatively large residual strain is observed for each cycle at 37 and 39 °C. The residual strain is rather small in the initial dozens of cycles at 31 and 33 °C. Moreover, the residual strain completely disappears over cycles and a pre-tensile force is generated at λ = 1 below the LCST. 

To further study the influence of λ_max_ on the mechanical properties of NIPA hydrogel under a cyclic load, the authors also conducted a mechanical cyclic test at λ_max_ = 1.4. Due to the critical maximum stretch λ_c_ of samples below the LCST being around 1.4, the breaking point of some samples falls within 1000 cycles in the tests of λ_max_ = 1.4. By analyzing the mechanical properties under a cyclic load at λ_max_ = 1.4, it is found that the evolution features of the stress-stretch curves are similar to those at λ_max_ = 1.2. It is demonstrated that the λ_max_ plays no significant role in the evolution of the stress-stretch curves over cycles. The detailed information on mechanical properties at λ_max_ = 1.4 is shown in Supplementary Information.

**Figure 7:**
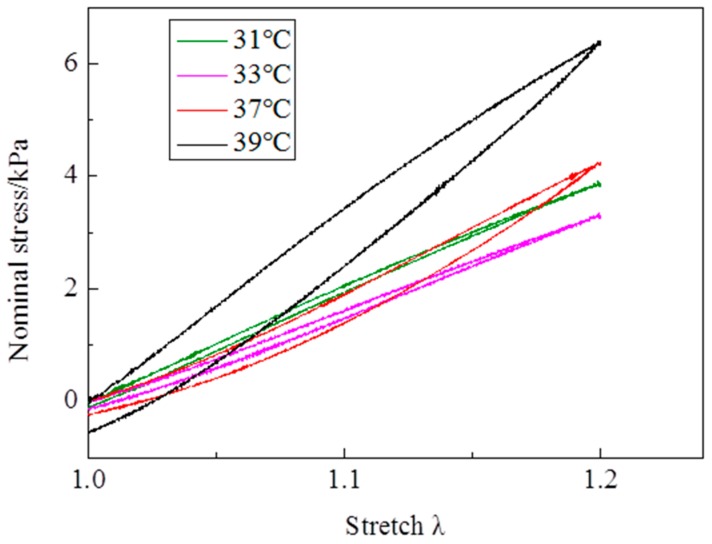
The load—unload stress stretch curves of the first cycle vary with temperatures at λ_max_ = 1.2.

**Figure 8:**
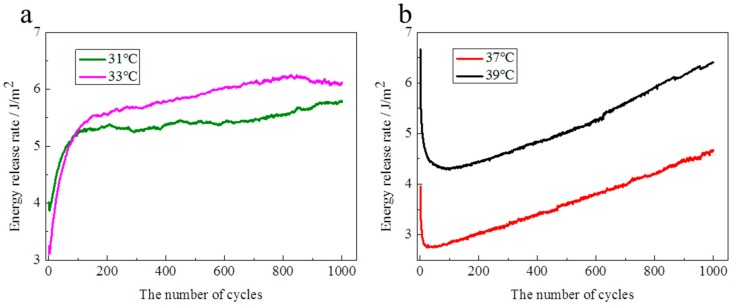
Evolution of energy release rate over cycles ((**a**), below the LCST; (**b**), above the LCST)).

Cyclic mechanical tests show that the mechanical hysteresis is significant above the LCST in the first cycle, while the hysteresis loop is rather small below the LCST in the first cycle (Figure 7). It is inferred that the mechanics of energy dissipation are different between the swollen state and the collapsed state during the tension process. Calculating the energy release rate by the load stress—stretch curve, the evolutions of the energy release rate over cycles are shown in Figure 8. Below the LCST, the energy release rate rapidly increases with cycles increasing at the initial dozens of cycles, and then slowly increases over cycles except for small fluctuations (Figure 8a). Over 1000 cycles, the energy release rate increases by 29% and 91% at 31 and 33 °C, respectively, in comparison to the value in the first cycle. Above the LCST, the energy release rate reduces with cycles increasing at the initial dozens of cycles, and then increases over cycles (Figure 8b). Moreover, at the increase stage, the energy release rate nearly linearly increases over cycles. The increased rate of energy release rate is 2.3 m J/m^2^ per cycle. Compared with the initial energy release rate, the energy release rate reduces by 29% and 35% at 37 and 39 °C, respectively, within 100 cycles, while the energy release rate increases by 20% and −3% at 37 and 39 °C, respectively, after 1000 cycles. After more than 1000 cycles, the energy release rate should be larger than the initial value at 39 °C, according to the evolution features of the energy release rate above the LCST.

**Figure 9:**
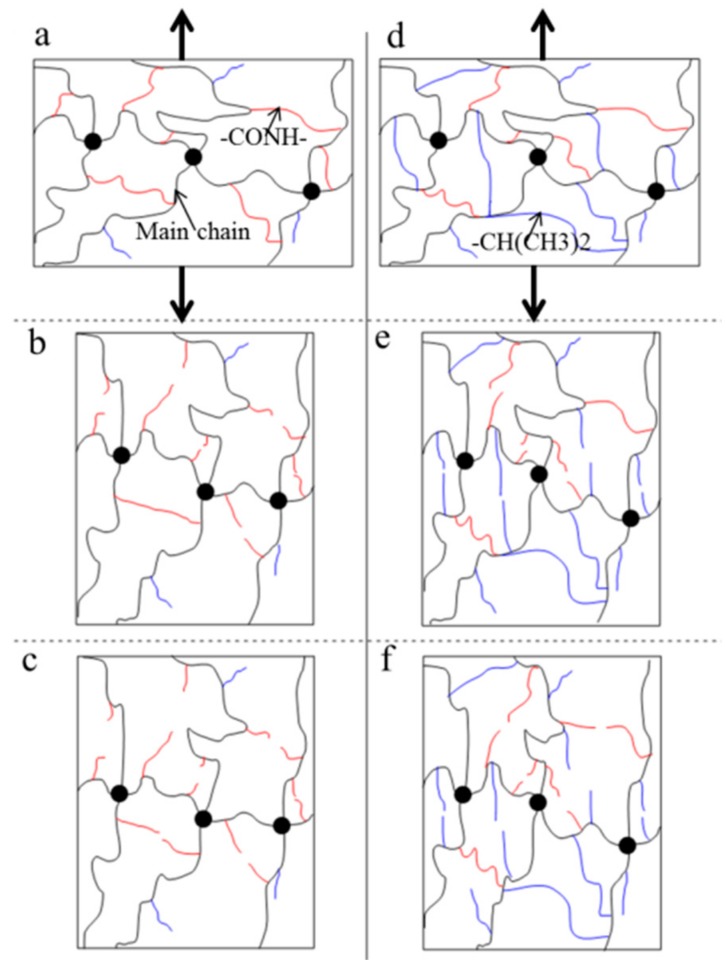
Schematic illustration of the internal fracture process and redistribution of the polymer network under a cyclic load. The network of NIPA gel is mainly composed of main chains in black curves by crosslinker (black dot). The red and blue curves stand for the weak chains generated by hydrophilic moieties (–CONH–) and hydrophobic moieties (–CH(CH_3_)_2_), respectively. (**a**,**d**) is the initial state of NIPA hydrogel before tension; (**b**,**e**) and (**c**,**f**) are internal fracture process and redistribution of polymer chains below the LCST above the LCST, respectively.

The NIPA hydrogel shows a toughening characteristic under a cyclic load (Figure 8). Furthermore, the toughening characteristics are different below the LCST and above the LCST, because the polymer network and water content have distinct differences. The responses of the polymer network under a cyclic load are also different at different temperatures. In particular, there are marked differences between below the LCST and above the LCST. The schematic illustration of the polymer network (Figure 9) is used to explain the mechanism of toughening under a cyclic load. In general, the polymer chains are in random distribution, the chain density in the swollen state (Figure 9a) is smaller than that in the collapsed state (Figure 9d). At the initial stage (Figure 9b,e), a large number of weak bonds are broken and main chains are redistributed along the uniaxial tension direction. Below the LCST, weak bonds are mainly composed of hydrogen bonding, while the weak bonds are mainly composed of hydrophobic moieties above the LCST. The hydrophobic moieties are stronger than the hydrogen bonding. Figure 8 indicates that the increased energy release rate by the redistribution of main chains along the tension direction is clearly larger than the reduced fracture energy by weak bonds broken below the LCST. Meanwhile, the increased energy release rate by the redistribution of main chains along tension direction is clearly smaller than the reduced fracture energy by weak bonds broken above the LCST. When the main chains finish the redistribution along the tension direction (Figure 9c,f), the increased energy release rate remains constant below the LCST. Hydrophobic associations are so strong that they are not destroyed during the expansion of the gel network in water [24]. Therefore, the energy release rate over cycles above the LCST maintains the increasing trend. The difference of the polymer network structure induced by temperature may be an important reason for the toughening mechanism under a cyclic load. The different toughening mechanism between below the LCST and above the LCST might be induced by the redistribution of main chains during the tension process.

## 5. Conclusions

NIPA hydrogel is attributed to a pendant isopropylamide group, which induces a change in the hydrophilic—hydrophobic balance as the temperature changes. In this study, the LCST for the NIPA hydrogel lies between 34.5 and 35.2 °C. The mechanical properties of NIPA hydrogel and effects of phase transition on mechanical properties are extensively investigated at temperatures of 31–39 °C under a tensile test. 

Under a monotonic load, the maximum nominal stress and maximum stretch above the LCST are larger than those of below the LCST. The Young’s modulus *E* is around 13 kPa at 31 °C and near 28 kPa at 39 °C. A dramatic increase of *E* values is observed as the temperature increases through the phase transition. The samples at a temperature around the LCST are easy to rupture, because of phase coexistent.

Under a cyclic load, NIPA hydrogels show toughening behaviors. Furthermore, the toughening characteristic is different between the swollen state and the collapsed state. This might originate from the internal fracture process and the redistribution of polymer chains during the tension process.

Quantitatively studying the effects of phase transition on mechanical properties for NIPA hydrogels may provide researchers with guidelines in designing the application in tissue engineering and regenerative medicine.

## Supplementary Materials

The supplementary materials are available online at http://www.mdpi.com/2073-4360/10/4/358/s1.
