# Supplementary material for: Phase Transition Effects on Mechanical Properties of NIPA Hydrogel
Source: Polymers (Basel). 2018 Mar 23;10(4):358. doi: 10.3390/polym10040358 (PMC6414852; doi:10.3390/polym10040358)
Supplement: Supplementary file 1 [file polymers-10-00358-s001.pdf]

SUPPLEMENTARY INFORMATION

The mechanical properties of NIPA hydrogel under monotonic load at the loading strain rate of 0.01mm/min is shown as following:

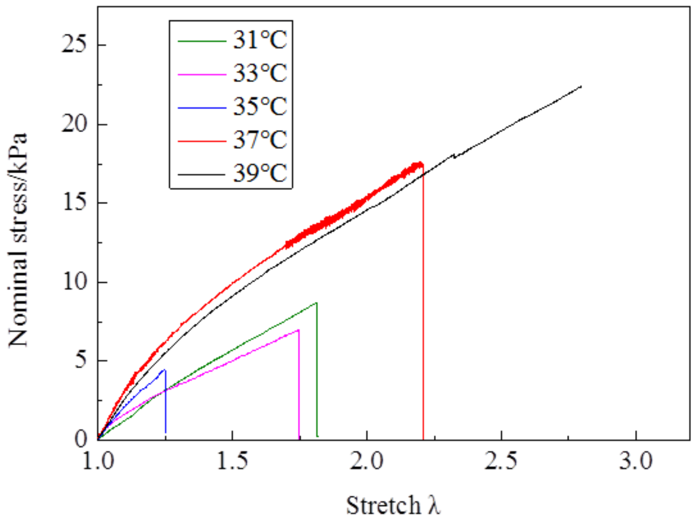

Figure. S1 Stress-stretch curves vary with temperatures

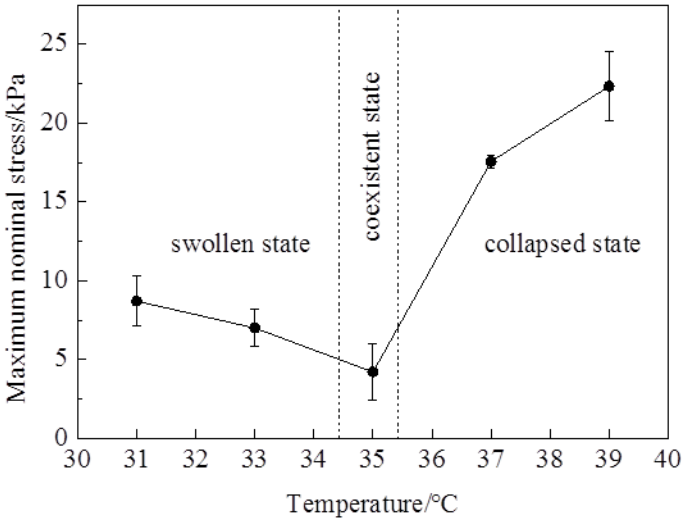

Figure.S2 Maximum nominal stress varies with temperatures

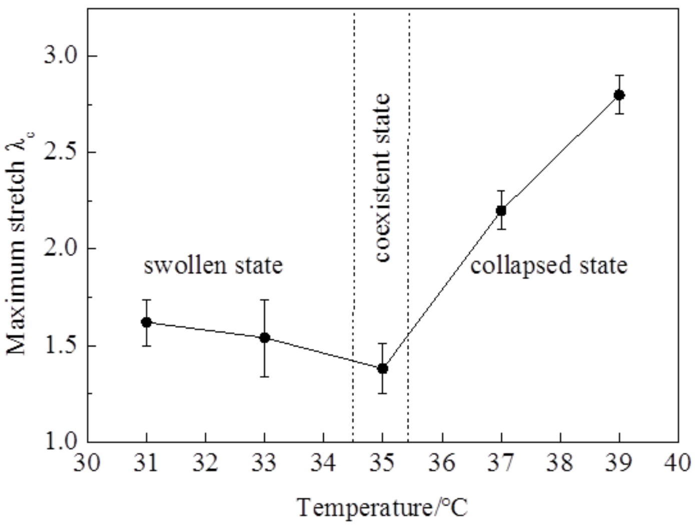

Figure.S3 Maximum stretch varies with temperatures

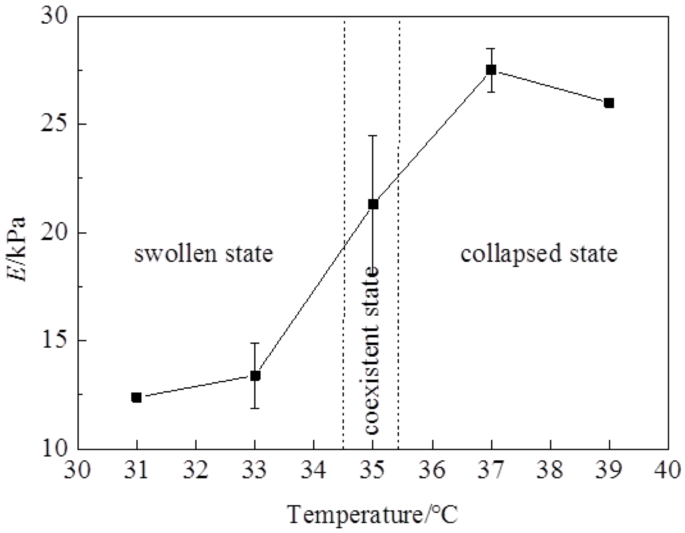

Figure.S4 The Young's modulus *E* varies with temperatures

The mechanical properties of NIPA hydrogel under cyclic load at λ_{max} = 1.4 is shown as following:

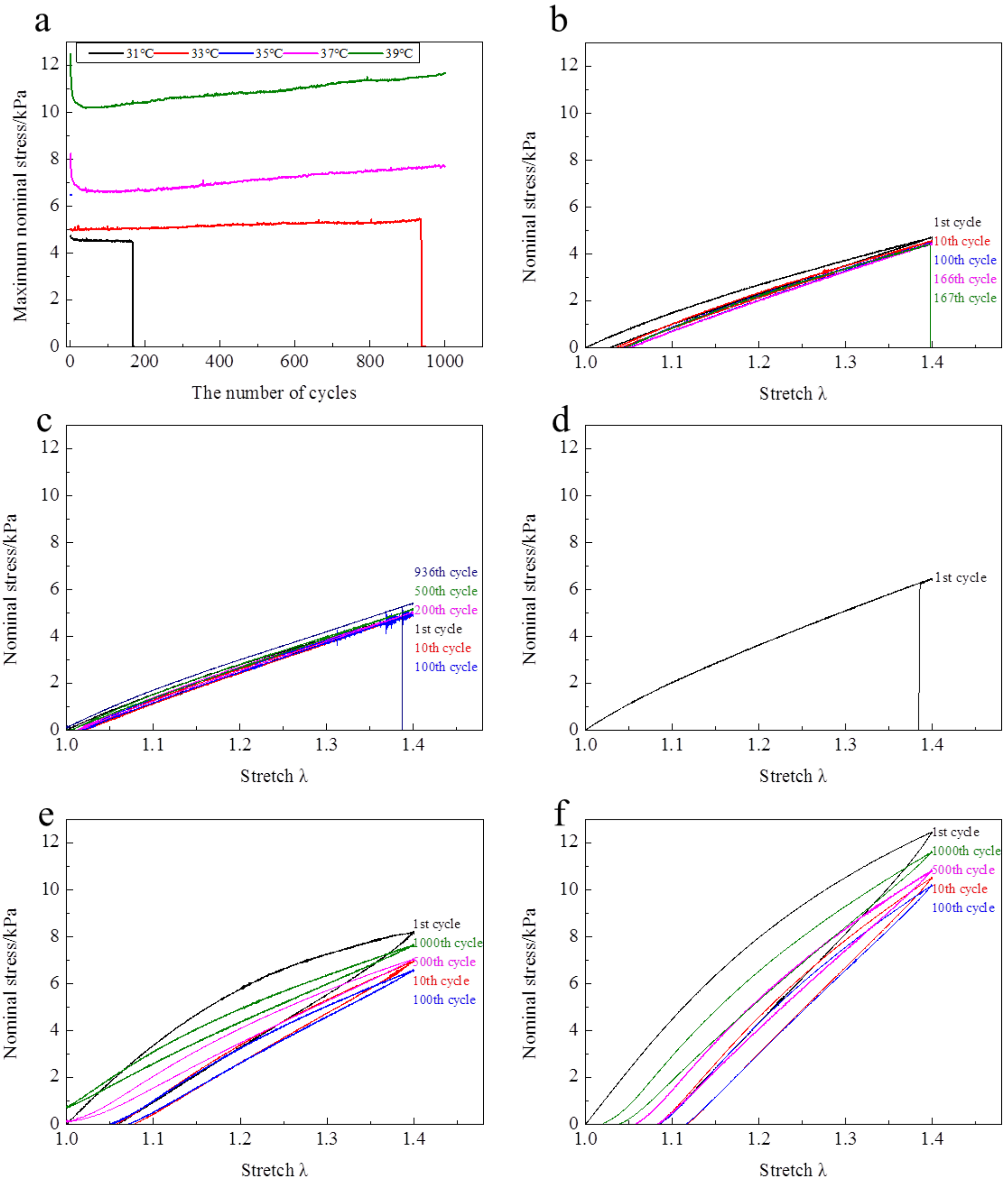

Figure.S5 Evolutions of stress - stretch curves over cycles at λ_{max} = 1.4 (a, evolutions of maximum stress in each cycle over cycles; evolutions of stress - stretch curves over cycles at 31°C (b), 33°C (c), 35°C (d), 37°C (e), and 39°C (f))

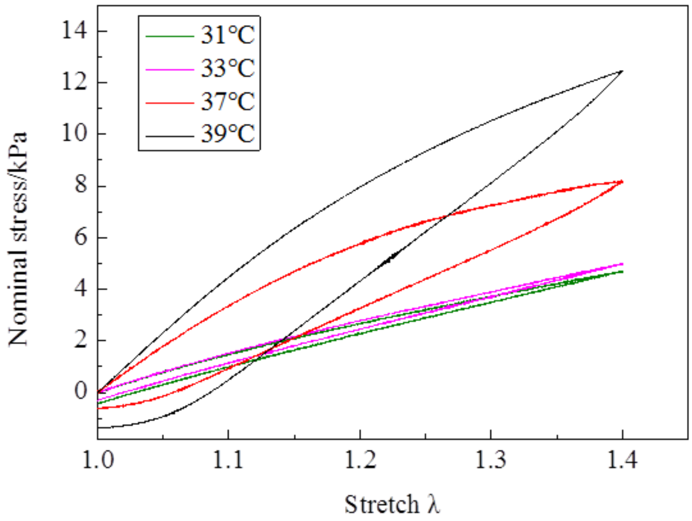

Figure.S6 The load - unload stress stretch curves of the 1st cycle vary with temperatures

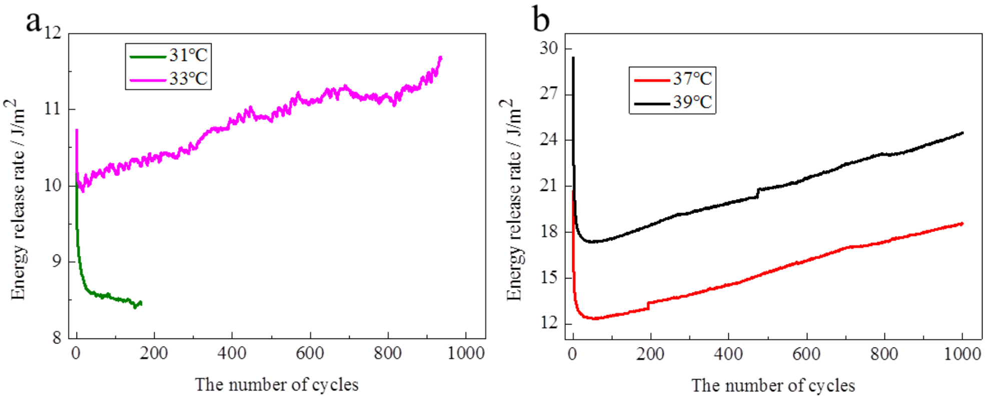

Figure.S7 Evolution of energy release rate over cycles (a, below the LCST; b, above the LCST)
